# Ghanaian views of short-term medical missions: The pros, the cons, and the possibilities for improvement

**DOI:** 10.1186/s12992-021-00741-0

**Published:** 2021-09-25

**Authors:** Efua Esaaba Mantey, Daniel Doh, Judith N. Lasker, Sirry Alang, Peter Donkor, Myron Aldrink

**Affiliations:** 1 Department of Social Work, University of Ghana, Legon, Ghana; 2 Centre for Social Policy Studies, University of Ghana (Now at Western Sydney), Sydney, NSW Australia; 3 Lehigh University, Bethlehem, PA USA; 4 Department of Surgery, School of Medical Sciences, Department of Maxillofacial Sciences, Dental School, College of Health Sciences, Kwame Nkrumah University of Science and Technology (KNUST), and Komfo Anokye Teaching Hospital, Kumasi, Ghana; 5 Medical and Surgical Skills Institute, Korle-Bu Teaching Hospital, Korle-Bu, Ghana

**Keywords:** Volunteers, short term medical mission, healthcare

## Abstract

**Background:**

Various governments in Ghana have tried to improve healthcare in the country. Despite these efforts, meeting health care needs is a growing concern to government and their citizens. Short term medical missions from other countries are one of the responses to meet the challenges of healthcare delivery in Ghana. This research aimed to understand Ghanaian perceptions of short-term missions from the narratives of host country staff involved. The study from which this paper is developed used a qualitative design, which combined a case study approach and political economy analysis involving in-depth interviews with 28 participants.

**Result:**

Findings show short term medical mission programs in Ghana were largely undertaken in rural communities to address shortfalls in healthcare provision to these areas. The programs were often delivered free and were highly appreciated by communities and host institutions. While the contributions of STMM to health service provision have been noted, there were challenges associated with how they operated. The study found concerns over language and how volunteers effectively interacted with communities. Other identified challenges were the extent to which volunteers undermined local expertise, using fraudulent qualifications by some volunteers, and poor skills and lack of experience leading to wrong diagnoses sometimes. The study found a lack of awareness of rules requiring the registration of practitioners with national professional regulatory bodies, suggesting non enforcement of volunteers’ need for local certification.

**Conclusion:**

Short Term Medical Missions appear to contribute to addressing some of the critical gaps in healthcare delivery. However, there is an urgent need to address the challenges of ineffective utilisation and lack of oversight of these programs to maximise their benefits.

## Background

Many countries in sub-Saharan Africa face the challenge of providing quality healthcare to their citizens as a result of limited resources. Successive Ghanaian governments, for example, have worked to increase the provision and quality of Ghana’s healthcare delivery. Irrespective of effort, the challenges remain for a government with inadequate resources. Short-term Medical Missions (STMMs) have become one avenue for extending healthcare delivery in countries such as Ghana.

Historically in Ghana, large numbers of people have engaged in short term medical missions yearly. They come primarily from High Income Countries (HICs) to provide medical care for a short period, usually four (4) weeks or less. These group include doctors, nurses, students, and non-medical volunteers. Yet there are no data available on the number of groups that arrive each year, where they work and what they accomplish. These activities are largely unregulated and unevaluated. It is therefore essential to understand their value and the challenges they present.

STMMs, also referred to as Short-term Experiences in Global Health (STEGHs) and Medical Service Trips (MSTs), have been variously defined as programs that last less than 8 weeks [41] or less than 6 months [24, 25], though the great majority last for 2 weeks on average [37]. Sponsored by thousands of organizations in the Global North governmental, corporate, academic, faith-based, and secular NGO’s. This growing phenomenon brings students and other volunteers to communities in the Global South to provide clinical services and supplies, research and education, and a variety of related support services intended to improve quality of life. As these programs are almost entirely unregulated, there are no data on their frequency, quality, or cost. Estimates have suggested that they may involve millions of volunteers and as much as $7.5 billion annually ([38], Paul Caldron, personal communication). One study found that half of program budgets is spent on airfare [5].

While many of these programs may be beneficial to host countries, they have come under increasing scrutiny, and serious concerns have been raised by scholars and practitioners across the diverse sectors involved. A growing number of publications have questioned the value of STMMs and proposed strategies for improving them (e.g., [3, 24, 25, 39, 42]).

Critiques center around possible harms to host countries and patients, including medical errors, non-alignment with local systems and priorities, cultural insensitivity, and the high cost compared to benefits. A lack of local direction and leadership, questionable ethics of practice, and whether they appropriately address community needs and provide long-term sustainable benefits are all widely questioned by scholars and health practitioners [24, 25, 28, 38]. Lack of evaluation and oversight make it impossible to determine the extent of benefits and drawbacks [24, 25, 31].

Studies of host partner organizations, community members, and staff in a wide variety of countries in the Global South, which pointed to some key challenges with managing volunteers and questions about their effectiveness [2]. For example, there are concerns about volunteers’ lack of cultural understanding, experience and preparation, attitudes of superiority, disrespect for local customs and practices, and imposing their own methods and opinions in ways that are inappropriate to the practice environment [20–22, 24, 25]. They also express a desire for greater continuity of care and better communication with volunteers, both in terms of language and in clarity of purpose. Some raise the possibility of foreign physicians competing with, or even replacing, locally trained professionals [18].

Studies also report satisfaction with the experience of hosting volunteers, including appreciation for the concern shown by visitors for underserved people, the “extra hands” they provide in severely understaffed situations, and the medical services and supplies they typically bring with them, their hard work, motivation, adaptability, dedication, high capacity to innovate, and their ability to teach specialized skills [22, 30]. Other research has confirmed that host organizations highly value volunteers with cultural humility and a willingness to learn from their hosts, share their technical skills, and provide training for local staff, as well as knowledge of the local language and culture [5, 6].

These criticisms of STMMs reflect many of the issues raised in critiques of humanitarian and development programs more broadly [13, 23, 35]. For example, as l’Anson and Pfeifer wrote about humanitarian aid, “… in most cases, NGOs and their supporters are deaf to the actual wants, needs, and desires – or, in other words, the agency – of those they are trying to aid.” This is a common concern with STMM’s, which are often driven by the needs of volunteers and the assumption that anything they offer is better than what communities already have.

Another key concern with STMMs, as with other kinds of humanitarian assistance, is the potential for undermining host country professionals. Free clinics and free medications offered by outsiders, often assumed to be superior in their clinical abilities even when they are not, can put local practitioners out of business or compromise their standing in the community. Similarly, free food aid has been seen to create conflict and put farmers out of business [34].

An underlying issue in these examinations of well-intentioned assistance is whether they perpetuate colonial and neocolonial relationships and feed what has been called the “White Savior Industrial Complex” [8]. Fassin and Pandolfi [14] cite the view that humanitarianism is the “nice face of a new colonialism” (p. 41). Global health discussions have also recently begun to focus on how to “decolonize” relationships between high- and lower-income countries [12].

One element of this “new colonization” is seen in the imbalance in power and benefits between researchers from high income countries and those in LMICs who often carry out research projects but do not get proper credit [27]. Additionally, advocates for Community-Based Participatory Research point out the lack of relevance and validity in studies of poor communities if they are not designed and directed by those communities [19, 44]. Accordingly, the study presented here, unlike almost the entirety of existing literature on STMM’s, does not come from scholars and practitioners in the Global North and is not based primarily on anecdotal experiences and surveys of volunteers [7]. While in recent years there has been increased attention to the viewpoints of host country staff [24, 25, 29, 37], these studies have been carried out almost entirely by outsiders, many of whom are affiliated with the programs they write about.

While the results of these studies are often insightful and do capture problems identified by host country staff, the inherent biases posed by social desirability and power differentials when researchers are from the Global North can pose threats to the validity of the results.

The purpose of this paper was to examine the benefits and harms of STMM’s from the perspective of Ghanaians, with the research designed and carried out by Ghanaian social scientists. It is likely one of the first such projects and thus of particular interest to health officials and scholars worldwide. The paper presents the results of interviews of host staff and health officials regarding their perspectives on the advantages and problems of short-term medical missions in Ghana. It also included analysis of the regulatory framework for STMMs in Ghana. This paper was part of a research project, sponsored by Lehigh University in Bethlehem PA, USA, which explored Host Country Views on Short-Term Health Volunteer Programs. Researchers in three countries that commonly host short-term programs designed and carried out the study; the first country examined was Ghana.

### Health care in Ghana

As of 2016, there were 6812 health facilities in Ghana, dominated by Community-Based Health Planning and Services (CHPS) (61.44%). CHPS is an entry-level healthcare system designed in 2000 to serve mostly rural communities and to respond to maternal health issues [45]. Health facilities with full hospital status (hospitals and district hospitals) account for about 6% of the health facility distribution, as shown in Table 1. The Table is arranged based on the distribution of the facilities within the health system.

**Table 1:** Health facilities in Ghana

Type of health facility	Number	Percentage
CHPS	4185	61.44
Health Centres	855	12.55
Clinic	1003	14.72
Midwife/Maternity	328	4.82
Polyclinic	34	0.50
Psychiatric Hospital	3	0.04
District Hospital	137	2.01
Hospitals	267	3.92
	6812	100

Source: Estimates calculated from Ghana Health Service [17]

In terms of doctors, according to estimates from the Ghana Health Service [17], there were 3365 doctors, with a doctor to population ratio of 1:8000 compared to the World Health Organisation’s recommendation of 1:1000 [46]. The evidence shows uneven doctor distribution across the country; the Upper East Region and the Western Region have the worst doctor to patient ratio of 1:25,878 and 1: 20,659, respectively. Similarly, the data shows that there are about 52,605 nurses in the country, with a nurse to population ratio of 1:542. The Western and Eastern regions have the worst nurse to patient ration of 1:728 and 1:704, respectively.

In Ghana, demand for healthcare is increasing as the population grows. The evidence shows annual out-patient attendance of 29,741,608 and in-patient admission of 1,532,845, representing about 53.9 admissions per 1000 populations in 2016 [17]. Malaria remains the topmost out-patient morbidity at 31%, and Upper Respiratory Tract Infections at 17%. Despite the significant strides made in reducing infant mortality in 2019 under-five mortality rates ranged from 31 to 66 deaths per 1000 live births compared to 51.7 for sub-Saharan Africa as a whole, there remain critical concerns in this area [43]. There are also increasing concerns over maternal mortality being high, although some gains have been made [40].

From this overview, it is established that the health context of Ghana presents opportunities for medical missions, particularly in the rural and deprived regions where there are clear gaps in personnel and health infrastructure. However, even though STMMs may deliver significant care to underserved populations, the growing critique by scholars and practitioners in the Global North raises important questions about how host communities and staff perceive these programs [21], the purpose of the present study. Therefore, the core research questions this paper seeks to answer are: What are views of Ghanaians of the benefits of STMM in Ghana (pros)? What challenges are associated with STMM in the country? And what is the Ghanaian regulatory framework governing STMM, and how widely is it enforced?

## Methods

### Research design

The study used a qualitative design, which combines the case study approach and political economy analysis. The qualitative research approach was utilised because the phenomena under investigation could be better explained through an in-depth understanding of the experiences and perspectives of the target respondents. The study relates to individual subjective realities and experiences of STMM with attention to the knowledge of the regulatory context of STMM in Ghana. The case study approach was relevant for exploring the contextual and experiential issues of short-term medical missions; it involves an in-depth investigation into a bounded unit within its real-life context to understand a larger class of similar units [15, 16, 47]. In the case of this current research, we considered the Ghana health system relative to medical missions as a bounded system and selected health sector institutions and respondents as elements within the case. The case study relied on in-depth interviews and was useful in examining Ghanaian stakeholders’ views of medical missions.

Given that medical missions are complex and involve different actors and different settings, a political economy analytical approach was also useful for unearthing potential institutional, policy and regulatory issues that can enhance or inhibit medical missions’ activities currently and in the future [1, 11]. The analysis process involved looking into the interview data to extract all institutions cited by respondents and examine their roles relating to STMMs. We complemented the data with a review of secondary information from policy, programme and legislative documents available or as cited by respondents.

### Data collection

**Fig. 1:**
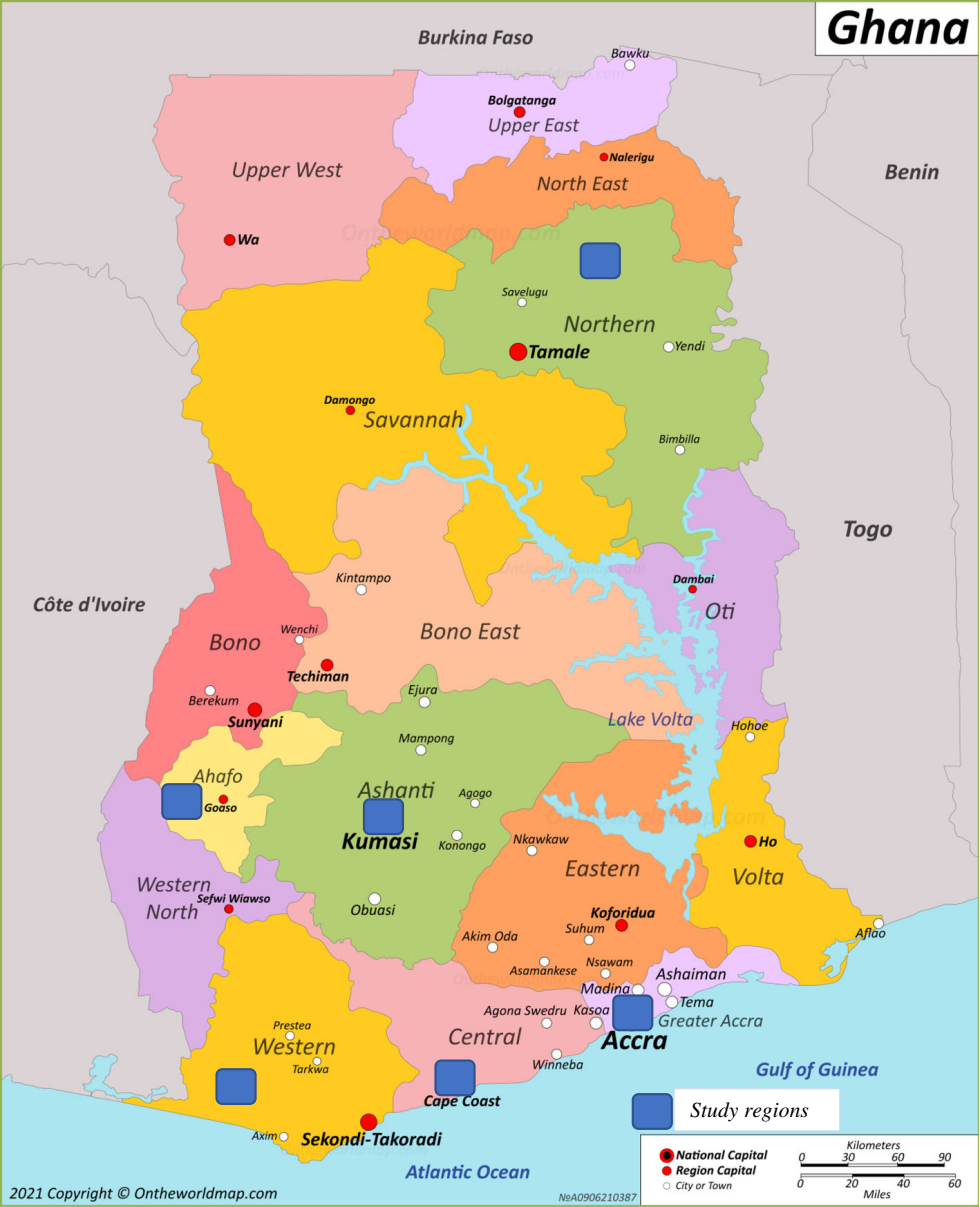
Map of Ghana showing the regions where research was conducted

The primary data collection for the case study involved in-depth interviews with respondents who were purposefully selected from healthcare institutions in six regions across the country: Greater Accra, Central, and Western along the coast, Ashanti and Brong-Ahafo in the middle of the country, and Northern region. The selection of the regions was based on consulting with known Ghanaian experts in public health regarding areas with a high concentration of STMM activities. This selection of regions provided a great diversity in population size and health care availability and were where many of the STMMS go. Fig. 1 shows the map of Ghana and the selected areas where the research was conducted.

The participants worked in a variety of institutions, including public and private/faith-based health service delivery outlets and government entities (regulatory bodies). The institutions were selected based on their experience relating to STMMs. Respondents were purposively selected based on their personal involvement in volunteer activities in the selected institutions.

Prior to the interviews, an invitation letter was sent to each identified institution from the categories mentioned above. The letter provided information about the research and requested an interview with one or two representatives who could provide answers to the core issues of the research. The selected institutions nominated their appropriate staff, and the interview sessions were organised accordingly. Where it was not possible to hand-deliver the letter, phone calls were made to the institutions. The project was approved by the Ghana Health Service Ethics Review Board and in line with the ethical requirements, all participants were asked to sign a written consent or provide a verbal consent prior to the interviews. The project was a low risk and did not pose any identified harm to the participants other than a potential discomfort answering questions.

In all, a total of 24 participants, comprising male and female. Eight (8) Medical Officers, seven (7) Nurses, seven (7) Administrators and two (2) Regulators, were involved in the study and were interviewed in person. The in-depth interviews focused on exploring respondents’ first-hand experiences of medical missions, particularly the nature of STMMs. They were asked about the countries where volunteers come from, types of activities undertaken by STMMs, the presenting challenges associated with STMMs activities and the critical benefits or outcomes of STMM. The interviews were conducted by the lead author and two trained Research Assistants. The interviews lasted for about 45 min on average and were conducted in English.

### Data management and analysis

An independent professional was recruited to transcribe the audio recorded interviews. The transcripts were spot-checked by the researchers to ensure consistency with the audio versions. The data were largely organised with the support of Nvivo 12 for Windows. Both deductive and inductive approaches to qualitative coding were used to facilitate the initial coding of the data. Regarding the deductive approach, some pre-determined themes were developed based on the theoretical context and the objectives of the current study. Concerning the inductive approach, some of the themes and their relevant codes emerged out of the data and were not initially developed. Some of the emerging codes were captured as direct concepts from the respondents’ words, and others were relabelled (given new concepts that retained the original meaning in order to capture similar meanings from other respondents). The codes were clustered to constitute categories (codes that collectively speak about similar issues) and labeled according to their respective themes, following the research objectives or as a new emergent theme. In some instances, code frequencies were derived based on the number of times participants spoke about an issue. The codes were initially developed by one researcher and were validated by the other researcher. Both researchers later met to harmonise the codes. The interviews were de-identified during transcription and given unique identifications to ensure privacy and confidentiality.

## Results

### Sending countries for STMM

To set the context for the discussion of STMM, we investigated the countries of origins of volunteers into Ghana. This analysis is based on how much information respondents could recall at the time of the field work and their experiences with STMM volunteers. Overall, most volunteers to Ghana came from the United States of America, and this was cited by 14 out of 24 respondents. Britain and Germany followed, with 6 (each) respondents citing them as sending countries. Canada (4 respondents) and other European countries including Czech Republic, Netherlands, Denmark, Finland, Switzerland (11 respondents) were also noted as important sending countries. China, Japan, Russia, and Haiti (1 respondent mention each) also sent volunteers to Ghana for STMM activities. It is important to observe that at least two respondents mentioned Ghana as a source of volunteers whom they have worked with. This provides some insight into internal STMMs activity in Ghana, where professionals travel to different parts of the country, particularly deprived areas such as the northern region to provide short-term medical care to deprived communities.

### Nature of activities of STMMs

In addition to our understanding of respondent’s perspective of where STMM volunteers came from, we examined the kind of activities or programs they implement. STMM volunteers coming to Ghana have diverse skills, and they include highly skilled medical professionals, medical students, and other support volunteers. Their programs were largely undertaken in rural communities and they addressed the medical conditions prevalent there. Table 2 is a summary of the core activities of STMM in Ghana. The codes reflect the intensity of the activities being described by participants and they have been arranged according to their frequency of mention. While some of the identified activities potentially overlapped, each has been made distinct from the other for the purposes of this analysis.

**Table 2:** STMM Activities in Ghana, organized by frequency of mention by respondents

STMM Activities/Program	Code freq	%
Community Outreach	48	28
Medical equipment and supplies	39	23
Surgeries and clinical activities	21	12
Capacity building for local staff	19	11
General consultations	16	9
Student field experience	12	7
Ward activities	7	4
Knowledge sharing	4	2
Research	2	1
Tourism	2	1
	170	100

Community outreach was the most cited program undertaken by STMM volunteers. This activity involves volunteers travelling to rural communities of Ghana to undertake short-term medical activities such as surgeries and general practice consultations. We used the concept” community outreach”, as a distinct descriptor characterised by activity involving travelling outside urban settings into rural communities upon arrival in Ghana. To reflect this understanding from the data, a respondent described activities of volunteers in these words:

*When volunteers come here, we go out to places where people do not have access to health facilities but only CHPS*^1^*Compound, and where they do not have access to tertiary facilities like the District and Regional Hospital. So, we go to those areas and stay there for about a week and we do consultations, provide drugs and other services, even dental services to them. So, we do not stay in health facilities but rather more of an outreach work in the rural areas (R3- medical officer).*

From the narrative above, it is obvious how volunteers, in collaboration with their Ghanaian counterparts, undertake outreach services in remote communities of Ghana. This highlights the demand-driven nature of STMM activities in a context where there are disparities in access to healthcare between rural and urban communities.

The second most significant STMM activity is the provision of medical equipment and supplies Respondents noted that volunteers often bring into the country medical equipment, from sophisticated machines to simple supplies such as gloves. Some of the narratives also show that volunteers bring medications. This is a significant contribution of STMM and fills an important gap in healthcare service delivery, according to respondents. Following the supply of medical equipment is the traditional role of volunteers in providing surgical services and other clinical activities. The data shows that areas of surgical activities include ENT^2^ services, eye, hernia, fibroid, cleft palate, and other complex surgical procedures. Furthermore, respondents cited capacity building for local staff as an important STMM activity in Ghana. In this instance, volunteers provided training activities either through workshops or on the job training and skills transfer aimed at retooling their local counterparts especially for some of the emerging surgical practices. This activity is reflected in a statement by a respondent, medical officer.

*At times they come on with skills which is quite different from us. As some of our colleagues observe them and then they would be picking their style of work and then they can also incorporate it into their system. (R22 – a medical officer from a regional hospital).*

Furthermore, some volunteers, according to the analysis, undertake general consultations in designated urban hospitals. The process involved having one on one interaction with patients for the purposes of making diagnoses and providing treatment. Of course, similar consultations were done as part of outreach programs where volunteers have to travel from the city to rural communities. Other volunteers, such as students and trainees, used the opportunity as field practice or internship Student volunteers who undertook medical missions in Ghana were sometimes trained by Ghanaian counterparts. There were other activities such as ward activities where volunteers helped ward nurses to take care of patients, administering medication and clean ups. These ward activities were mostly performed by less experienced volunteers or those under training. In addition, we found research as one of the activities mentioned by some respondents. This is how one expressed the notion of research as an activity of STMM.

*From my experience, I think some come here because of the research aspect of it. They are able to get data and they are able to see the conditions physically and then be able to get the data to be used for research purposes. (R17 - nurse from an urban hospital).*

Tourism or sight-seeing also emerged as one of the activities undertaken by STMM volunteers. In some cases, tourism was planned and integrated into the work of volunteers, and in other cases, it was a way to unwind after working hard for two or more weeks. The idea of tourism was confirmed by a respondent who said:

*The last time they [STMM volunteers] came, we took them to Kakum National Park after the surgeries. They said they wanted to go to Mole National Park and other places but the days they spent in my facilities were sometimes 14 days … and the rest of the days are for sightseeing before they go back to their home countries (R1- a hospital administrator).*

### Challenges of STMM activities in Ghana

We examined the challenges associated with STMMs from the perspectives of respondents. Overall, we found 9 challenges (74 codes in total) cited by respondents as shown in Table 3. The most frequently cited challenge of working with volunteers on medical missions is the issue of language barrier (21%), particularly since most STMMs activities are concentrated in rural communities, where community members hardly speak any English. Therefore, from the narratives, there is a limit to how well volunteers can make connections with patients, and this affected the diagnosis process as explained by respondents. The point was typified by a respondent who said that:

*Some of the difficulties are that most of them [volunteers], because they do not understand the language, they really do not do a lot of physical examination on the patient, and then they just see the case. They are not able to speak to the patient about the fears and problems that might come up with the operation. They are just interested in cutting so they do not talk mostly to the patient (R17 – a theatre nurse).*

From the above narrative, some patients appear to be disadvantaged when it comes to volunteers. Although the data show the use of interpreters in certain cases, the interpreters are usually not professionals, which might increase the likelihood of misunderstanding.

**Table 3:** Challenges of STMM organized by frequency of mentions by respondents

Challenges of STMM in Ghana	Code Freq	%
Language as a challenge for volunteers	16	21.6
Undermining local knowledge and expertise	12	16.2
Pressure on host to provide support services	10	13.5
Fraudulent qualifications, poor skills and lack of Experience	9	12.2
Making wrong diagnosis	8	10.8
Unusable medical equipment and supplies	8	10.8
Personality conflict	6	8.1
Professional inferiority complex	3	4.1
Frustration due to poor equipment	2	2.7
	**74**	**100**

The second most cited challenge of medical missions by respondents is the tendency of volunteers to undermine local knowledge and expertise. This was cited in 12 cases. Some respondents related occasions where volunteers have ignored the expertise of their local counterparts, considering them as inferior and to an extent refusing to comply with local protocols.

*This hospital has collaborations where people had come and want to behave as if they are the ones who know and everybody does not know anything. So those collaborations had stopped over time (Doctor).*

The narratives suggest that patients who were seen by volunteer practitioners also believed that the volunteer health professionals were superior and better qualified than their Ghanaian counterparts. This was demonstrated in circumstances where patients insisted on seeing the volunteers who had treated them, even after they had left the country.

In contrast, one hospital administrator responded to a question about whether the volunteers are respectful by saying,

*How can they show disrespect? I have my facility and you walk into it to show me disrespect then I would show you the exit [Laughing…] you understand {I: Yes} so when they come they respect us because they are coming to beg for us to accept them to come and work in our facility. There is no way they can disrespect us. That is out of the picture completely.*

Another commonly cited challenge of STMMs, as shown in Table 3, is pressure on hosts to provide support services for volunteers (#10). Host institutions noted that often there were unanticipated costs to them for hosting volunteers, ranging from hotel accommodation to transport and entertainment. The evidence shows that host institutions do extra work to organise STMM activities successfully. One respondent confirmed this in the following words:

*They [volunteers] always mean more work and more cost. I told you that we host them in a hotel, you must be prepared to feed them as well and this comes with a cost, but we see it as part of our Corporate Social Responsibility. Sometimes hosting volunteers means more work, and costs may include the cost of preparing for them, hosting and organising programmes (R1 – hospital administrator- Ghana).*

The statement above is indicative of some of the contributions Ghanaian health professionals and institutions make towards STMMs, mainly in the areas of accommodation, food, transportation, and entertainment. They also noted the training and oversight of volunteers.

Fraudulent qualifications, poor skills and lack of medical experience form another set of challenges of STMM in Ghana, representing 12.2% of the code distribution. The evidence shows that in some instances, volunteers present themselves as experts when in fact, they do not have the requisite qualifications to practice. This was referenced in the statement below:

*Sometimes some volunteers came in parading as specialists and they turned out to be students and they were now learning. Once you find out that, you need to be able to stop them. I know the Medical and Dental Council is being blamed for being hostile to people outside, but they do this because they need to protect the public and guide the profession. So now they are quite serious and strict about who comes into volunteer (R6- medical officer from a municipal hospital).*

Furthermore, it emerged from the analysis that, given the context of disease conditions in Ghana and the lack of diagnostic machines that are more widely available in high income countries, some volunteers make wrong diagnoses. This is based on the fact that some of them do not understand the nature of some diseases such as fibroids and tropical diseases like malaria and typhoid in Ghana. This concern was seen in 10% of the total codes on the challenges of STMMs. In two cases, respondents reported that some patients seen by some volunteers soon return to the facility due to recurrence. For example, a respondent said:

*I must be frank with you because I told you that sometimes they [volunteers) manage cases and the patient comes back… so the patient will go for 2-3 years and come back that the fibroid has come back. Are you getting the point? Those are the challenges that we have. You have people coming back to complain that “oh I came here last year and the white guys removed my fibroid but it seems it has come back again.” You check the scan and you see that it is true there are more fibroid there but if you the Ghanaian doctors do it… because it is a Ghanaian and an African problem [presuming that the fibroid would not have returned if a local Doctor does the operation] (R1- hospital administrator).*

Another respondent notes:

*“…different countries have slightly different ways of treatment. So sometimes we have misunderstandings. But we in Ghana know a lot about typhoid fever; they do not know about typhoid fever so whenever they come here, it is one disease, we have problem with them. In other words, we think that they do not know that area and therefore they should probably not be operating on such illness. They should leave such cases for us. Diseases that are not common in their country should be handled by us. So sometimes we have difficulties getting along with them in that area (R16 – medical officer).*

Furthermore, other challenges cited in the data include unusable medical equipment and supplies (10.8%), personality conflict between volunteers and local counterparts (8.1%), inferiority complex on the part of volunteers when they cannot perform specific local treatment (4.1%), and expressions of frustration on the part of volunteers due to poor or lack of equipment for diagnostic purposes. On the non-usability of medical equipment, a respondent indicated that:

*Some of the equipment goes through clearing only to realise that they would not suit your purpose. All these in the name of “donation” or “help” to the hospital. Meanwhile you have to clear and transport them to your warehouse at a cost. You know equipment could come in with manuals that no one could translate. These are some of the challenges that we have encountered. Some of these things are duly reported to the ministry (R4 – hospital administrator).*

Another administrator recalled a similar circumstance:

*I remember (the organization) sent us a 40-footer container full of medical equipment…but they did not ask us before they brought that equipment. They came to observe and then we were there, and they called us that can we go to the port because a container is arriving. They send us the waybills and items in the container to go and clear it.*

On frustration on the part of volunteers, a respondent noted:

*Sometimes they get frustrated because they do not have the equipment and access to laboratory facilities because sometimes, they want to do certain tests. But for us we have been trained to take a very good history and examine the patient even without doing laboratory test and with that information we can give treatment but they cannot. So sometimes they get frustrated and cannot even work (R3 – medical officer).*

### Benefits of STMM activities in Ghana

Despite the challenges associated with STMM activities in Ghana, STMMs have made some important contributions to health care delivery in Ghana, especially among poor and rural dwellers. Some of the key benefits of STMM are presented and discussed below.

**Fig. 2:**
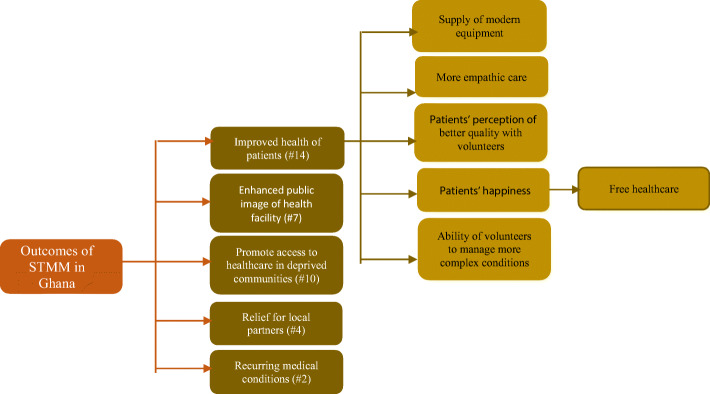
Benefits of STMMs showing the number of times references were made (code frequency).

As shown in Fig. 2, five main benefits emerged from the data. The most frequently cited positive outcome of STMMs relates to improvement in the health of patients. This was cited by 14 out of 24 respondents on 23 occasions and demonstrates that patients who had been seen by volunteers had experienced significant improvements in their health.

Improvements in the health outcomes for patients were driven by several related factors, some of which include the use of modern equipment and supplies by volunteers who come along with their own equipment, and a perception of an empathic approach to treatment by volunteers. For instance, some cite the ability of some volunteers to manage very complex medical conditions such as surgeries which contributed to improving the health of patients.

A nurse cited several of these benefits as follows:

*Most at times they come with certain equipment we do not have them and do not even know how to use them. They come to train us on how to use that equipment. Then they end up leaving that equipment for us.*

Of critical note is the happiness of patients about volunteers, which is often due to the provision of free medical care. It is noted that most services provided by STMM were free. Since the pressure of payment for medical treatment is reduced when volunteers come, the health conditions of patients improve faster, according to the narratives. Thus, patients believe that since they cannot afford healthcare that they need, their health condition deteriorates, but it improves when volunteers bring free services and equipment.

The second most frequently mentioned outcome of STMM is the promotion of access to healthcare for deprived and rural communities in Ghana. This was cited on 14 occasions by 10 participants who were mostly from rural areas. As noted from the literature, one of the healthcare challenges facing Ghana is the issue of access to quality healthcare and the unequal distribution of health professionals and infrastructure [17]. STMMs, therefore, help fill this gap and create opportunities for quality and often free healthcare to benefit poor and rural dwelling people. For instance, community outreach was highlighted as the most significant activity of STMMs. A respondent indicated that:

*I think they really do a lot for us because when you go to the villages that is where you realise that our health system is very bad. Those in the rural areas do not really have access to what a normal citizen should have. So, most of them stay there with so many diseases they are not even aware of. I think it really helps because sometimes we go there and find out cases especially with the children so we help to refer them to hospitals, tertiary centres and all that for surgeries. So, if we do not go how would these people know that they have a problem? (R3 - medical officer)*

The analysis further found that STMM activities contribute to enhancing the public image of health facilities (cited by 4 respondents on 7 occasions). Respondents explained that when volunteers come to their facilities, this attracts patients and their families, and as they receive treatment, they form positive images of the facility. An administrator in a hospital indicated:

*And then it also gives the hospital good image - that is it enhances our goodwill because if people are coming for supposedly free services people think that they get the quality they expect at a lower cost (R10 – hospital administrator).*

Finally, as expected, it emerged that STMM serves as a source of additional capacity for local counterparts to ease the pressure of healthcare provision temporarily (cited by 4 respondents). Respondents noted that volunteers augment their staff numbers and share knowledge and skills with them which is a great source of support.

### Regulatory frameworks for STMM

*I think the problem with these our volunteers is that they are scattered, not well coordinated from above, so everyone is doing his own thing in his own way but if it was such that there was a coordinated unit, maybe in the Ministry of Health (MoH) or the Ghana Health Service… (R5 - medical officer/surgeon).*

The analysis of the regulatory frameworks for STMM focused on institutional and legislative arrangements for STMM activities in Ghana. The analysis is based on a review of government documents and some of the insights provided by respondents. We asked all participants about their knowledge of laws or policies that regulate the activities of STMMs. Apart from the two respondents from regulatory institutions, there was no indication from participants that they had any knowledge of a specific legal or policy framework for STMMs. The context of regulations for STMM activities is summed up in the above quotation from a respondent – “everyone is doing their own thing”, which is indicative of a lack of coordination and enforcement of regulation.

However, it is our understanding from respondents from the regulatory institutions that since STMMs operate within the broader healthcare context of Ghana, the legal and policy context of healthcare affects them as well. Some of these includes mandatory registration and licensing to practice in the country.

The Ghana Health Service’s Codes of Conduct makes it mandatory for all health professionals working with them to be registered and to remain registered with their Professional Regulatory Bodies. The Health Professions Regulatory Bodies Act, which was developed in 2013, also set up health regulatory councils, including the Ghana Medical and Dental Council, with the mandate for ensuring standards, training, registration and regulation of medical and dental professionals. The Ghana Nurses and Midwifery Council, the Ghana Pharmacy Council, and the Allied Health Professions Council are other regulatory agencies with a mission to secure, in the public interest, the highest standards of training and practice. In doing so, all of the Councils have the ability and authority to provide temporary licenses to health professionals who wish to practice in Ghana for 3 months or less.

Of interest is the Medical and Dental Council’s position statement on regulation of medical practice as noted below:

*It is against the law to practice in Ghana without being registered with the Medical and Dental Council of Ghana; it is also unlawful to employ and engage the services of a practitioner who is not registered with the council*.^3^

As one interviewee from a regulatory council noted, it is up to the host organizations to enforce the rules for the benefit of their communities:

*NGOs on the ground are the people who realise that this is a community, and these are the needs, so I can invite these people to meet those needs (R18 – Someone from a professional and regulatory council).*

Many different governmental and organizational actors are potentially involved in STMMs or could be part of the regulation of such activities. Some provide regulatory and oversight roles through registration and licensing of health professionals. Others provide the policy context and health infrastructure for STMM activities, including hospitals and clinics. Even the Ministry of Foreign Affairs contributes indirectly by facilitating visas.

## Discussion

The study examined Short-Term Medical Missions (STMMS) in Ghana, particularly their activities, benefits, and problems. Ghanaian participants from around the country indicate that STMMs there, as in other countries, most frequently conduct outreach to rural communities and provide equipment and supplies [24, 25]. Less frequently mentioned in this study, despite being noted in the literature as most desired by host country staff [37], are information sharing and building capacity of local staff as key activities. These findings support the critique of STMMs that they often do not have any lasting impact.

Additionally, studies have found many gifts of equipment and medications to be inappropriate and even burdensome [9]. This was confirmed by a number of participants who had to go to considerable trouble and expense to get equipment they had not requested out of customs.

Community outreach is crucial for addressing the rural-urban health need gap since rural areas have limited healthcare personnel. Yet it is unknown how much of this gap is filled by STMMs, which arrive infrequently and are not evenly distributed in rural areas. Where they do exist, participants cited five major benefits-- improved health service delivery and positive outcomes for patients who received support through STMMs, enhanced public image of health facilities leading to revenue generation, relief or additional capacity for local partners, and the provision of medical equipment and supplies to facilitate health service delivery.

Despite positive benefits, participants cited difficulties that compromise the effective utilisation of STMMs. The most frequently mentioned challenge was language barriers. As explained by Meuter et al. [32], communication is central to the diagnostic process. Effective diagnosis is a combination of interviews and physical assessments, and if there is a barrier to doctor-patient communication, the likelihood of error is high. Therefore, some respondents felt that the problem of language in STMMs was a cause for concern [10]. A similar finding about the relevance of language and culture for medical missions was made by Chiu et al. [7] in a study on the perceptions and efficiency of short-term medical aid missions carried out by Taiwanese health professionals. And while other studies have noted the problem of language gaps [16, 22], none have indicated it to be the leading challenge.

The second most frequently cited challenge is that the volunteers may undermine local professional’s standing. This is seen in the observation that some patients who were seen by volunteer practitioners believed them to be superior to and better qualified than their Ghanaian counterparts. This finding is similar to an observation made by Nouvet, Chan, and Schwartz [33] in their study of medical missions in Nicaragua. The lack of respect for the expertise of host country practitioners, arising from the ignorance and biases of visiting volunteers as well as the colonialized attitudes of patients, has been cited elsewhere [29] but is more prominently mentioned here. We found less evidence of direct competition with local practitioners than cited in some writings, due to the lack of private practitioners in the rural areas.

Yet Ghanaian participants often emphasized the value of their own experience and ability to provide care with limited means and for conditions they know better, in contrast to foreign volunteers. Notable are instances reported of convincing the visitors of their capabilities. Thus while the issue of lack of respect that is often cited in other studies [26] is apparent here as well, there were a number of expressions of pride at changing those attitudes, a valuable finding not noted elsewhere.

For example, a surgeon responded to the question about respect as follows:

*Do they feel that they know too much or they are coming with exceptional skills? May be when they are coming they will feel so but after sharing knowledge then they realize that we know what we are about.*

Perhaps the most striking example comes from a hospital administrator:

*They tell them they are going to Africa and everyone want to go to Africa so they join them to see the black monkeys here [Laughing…] In fact, they come here to see that we are no monkeys after all. You understand. When they come, they marvel at the level of expertise of the Ghanaian doctors and nurses. Sometimes our guys teach them some of the things that needed to be done.*

While this comment prompts discomfort, it is perhaps the most obvious example of the advantage of the study being carried out by Ghanaians. Surely the comment would not have been expressed in this manner to an American, and yet it is a very powerful evocation of the many positive as well as troubling dimensions of STMMs. Interviewers noted that participants often switched to the local language to answer questions asked in English, even though the latter is Ghana’s official language and all participants were fluent in English. This tendency, along with side comments such as “you understand”, as seen in the quote above, indicate a level of openness and comfort that is necessary for research.

Another important contribution of this study is our analysis of Ghanaian regulations governing medical practice, as well as our questioning of study participants about their awareness of these regulations. The fact that there are requirements for approval for anyone seeking to practice in Ghana, and yet none of the staff interviewed were aware of them, reveals an important factor contributing to the problems with STMMs not addressed in other studies.

**Fig. 3:**
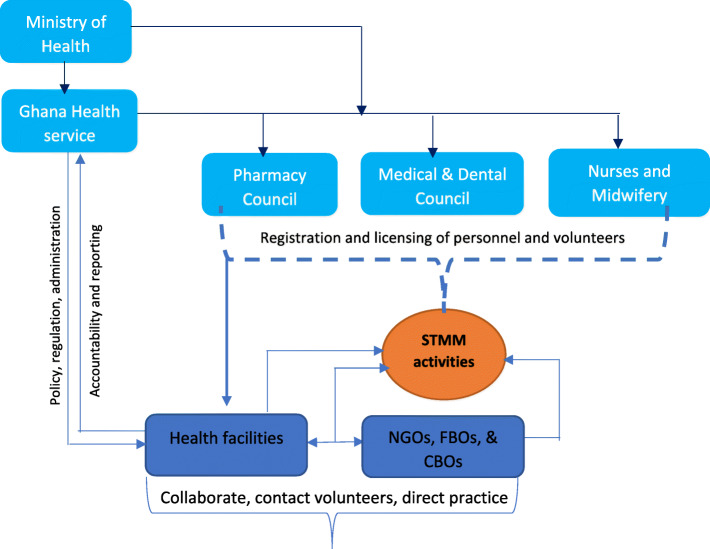
Conceptualization of institutional regulatory structure of STMM

Based on the narratives of some respondents, juxtaposed with the respective legislative and regulatory arrangement, we conceptualise what the institutional regulatory structure for STMMs could be in Fig. 3. The figure shows the Ministry of Health as the apex regulatory institution providing both legislative and policy context for STMM. Beneath Ministry is the Ghana Health Service (GHS) with a mandate to coordinate, deploy staff, and implement healthcare programs. The GHS has an oversight role over the three regulatory institutions of healthcare including the Pharmacy Council, the Nurses and Midwifery Council, and the Medical and Dental Council. These regulatory institutions have the mandate to register and license healthcare professionals including Volunteers. Below the authority structure are the healthcare facilities, health-based not-for profit and faith-based organisation with interest in health care. These institutions mostly have direct relationships with STMM. As shown in the conceptual regulatory structure, there is a weak link between the major regulators and STMM activities and this raises concerns for the enforcement of regulations.

It is mandatory for STMM volunteers to register with the regulatory Councils prior to their temporary practice. It is also mandatory for local partners to ensure that their volunteer visitors are properly registered. However, most local practitioners did not indicate an awareness of such provisions. And we are unable within the current data to ascertain what proportion of medical and dental volunteers actually do register with the Council. As alluded to by a respondent – “everyone is doing their own thing”, there are indications of some, or perhaps most, volunteers avoiding the public regulatory system. The regulators confirmed that they sometimes register and license volunteers when an application is made to them. However, they had well-founded concerns about volunteers possibly escaping the regulatory system.

## Conclusions

The challenges posed by international volunteers are often overlooked by host country officials because the activities largely take place outside the regular healthcare delivery system. Sponsoring organizations in the Global North are also not subject to regulations, and visiting volunteers assume that oversight is not needed because whatever they offer must be better than the “nothing” they assume they will find [24]. Competing priorities leave regulation of STMMs ignored [25].

The results of this study lead to the recommendation that challenges of STMMs should be given attention in order to reduce the ineffective utilisation of the programs. For instance, healthcare professionals from sending countries should have the requisite skills, knowledge and experience to meet the needs of the identified country and community, as determined by those communities. Regulatory agencies in Ghana are charged with evaluating qualifications and should do so for short-term volunteers. This will require better communication of the rules to host organizations as well as to sending organizations.

Sending countries or mission organisers should involve host countries in the planning process to clearly identify the health needs of the host communities. Also, the institution and councils responsible for health services should be able to coordinate and oversee the activities of STMMs to avoid unethical practices that could lead to abuse and to ensure equitable distribution of their activities to areas of need [36].

Despite the availability of these regulatory institutions, their level of control and coordination of volunteers who practice in the country is unclear. Therefore, as an example, the basic data on the number of volunteers per year and their respective originating countries are not available due to the lack of coordination. Most striking is the finding that none of the Ghanaian health professionals interviewed were aware of the regulations.

There is a need for all stakeholders in the health sector to have a dialogue about the future of STMMs so that Ghana can position itself to maximise the benefits and reduce the risks. A number of the personnel interviewed evidenced confidence in their own abilities relative to outsiders and the experience of rebalancing relationships in a more equitable way. Finally, this study supports the need to reduce the power imbalance between healthcare professionals from the sending countries and host country and to foster healthy relationships through mutual partnerships. These recommendations for more productive and ethical partnerships are reflected in the recently published Brocher Declaration [4], and wider dissemination may be useful in fostering conversations about achieving improvements.

Future research might well focus on the process by which regulations are created and enforced in host countries, and the incentives to adhere (or not) to those regulations. It would also be of great interest to investigate the advantages and disadvantages of the suspension of STMM during the pandemic and consider ways in which lessons learned during this time can be applied to changed policies and practices going forward.

## Data Availability

Data would be available upon request.

## Footnotes

1 Community-Based Health Planning and Services

2 Ear Nose and Throat

3 http://mdcghana.org/registration/
